# A methodology for distinguishing divergent cell fates within a common progenitor population: adenoma- and neuroendocrine-like cells are confounders of rat ileal epithelial cell (IEC-18) culture

**DOI:** 10.1186/1471-2121-6-2

**Published:** 2005-01-18

**Authors:** Phillip V Gordon, Jessica B Paxton, Nena S Fox

**Affiliations:** 1 Department of Pediatrics, University of Virginia, PO Box 800386, Charlottesville, VA 22908, USA; 2 Department of Microbiology, University of Virginia, PO Box 800734, Charlottesville, VA 22908, USA

## Abstract

**Background:**

IEC-18 cells are a non-transformed, immortal cell line derived from juvenile rat ileal crypt cells. They may have experimental advantages over tumor-derived gastrointestinal lineages, including preservation of phenotype, normal endocrine responses and retention of differentiation potential. However, their proclivity for spontaneous differentiation / transformation may be stereotypical and could represent a more profound experimental confounder than previously realized. We hypothesized that IEC-18 cells spontaneously diverge towards a uniform mixture of epigenetic fates, with corresponding phenotypes, rather than persist as a single progenitor lineage.

**Results:**

IEC-18 cells were cultured for 72 hours in serum free media (SFM), with and without various insulin-like growth factor agonists to differentially boost the basal rate of proliferation. A strategy was employed to identify constitutive genes as markers of divergent fates through gene array analysis by cross-referencing fold-change trends for individual genes against crypt cell abundance in each treatment. We then confirmed the cell-specific phenotype by immunolocalization of proteins corresponding to those genes. The majority of IEC-18 cells in SFM alone had a loss in expression of the adenomatous polyposis coli (APC) gene at the mRNA and protein levels, consistent with adenoma-like transformation. In addition, a small subset of cells expressed the serotonin receptor 2A gene and had neuroendocrine-like morphology.

**Conclusions:**

IEC-18 cells commonly undergo a change in cell fate prior to reaching confluence. The most common fate switch that we were able to detect correlates with a down regulation of the APC gene and transformation into an adenoma-like phenotype.

## Background

In the last quarter century, more than five hundred published manuscripts contained experiments using the popular rat crypt cell lines IEC-6 and IEC-18 originally established by Quaroni and Isselbacher in 1978 and 1981 [1,2]. These lineages have stood the test of time as being two of the most reliable and phenotypically well-preserved gastrointestinal cell lines to date. In our lab, we utilize the IEC-18 lineage because it is more robust in serum-restricted conditions and, in our experience, it is more suitable for correlation with distal intestinal crypt and enterocyte physiology than cell lineages derived from colonic tumors.

One of the more exciting aspects of IEC-18 cells is their capacity to retain the crypt cell phenotype as well as the continued potential for differentiation/maturation into enterocytes [3-5]. However, as we have worked with this lineage over the years, the rare but unmistakable morphology of an occasional neuroendocrine-like cell, the suspicious frothiness of a possible paneth-like cell and the rare giant vacuole of a goblet cell precursor have prompted us to wonder how prevalent spontaneous differentiation towards other lineages and/or transformants might be (especially in serum-restricted conditions).

This is a fairly crucial experimental question because serum-restricted conditions allow the tightest experimental rigor but may drive IECs towards altered cell fates, potentially introducing uncontrolled experimental confounders. Until recently, we did not have the necessary tools with which to determine more subtle epigenetic divergences in IECs. Our current investigation uses a novel strategy to demonstrate that spontaneous cell fate divergence is not only highly prevalent in IEC-18 cells, but it is fairly uniform and therefore a predictable confounder for gene array studies in this cell line.

## Results

### Initial characterization of IEC-18 culture heterotypy using immunolocalization

**Figure 1:**
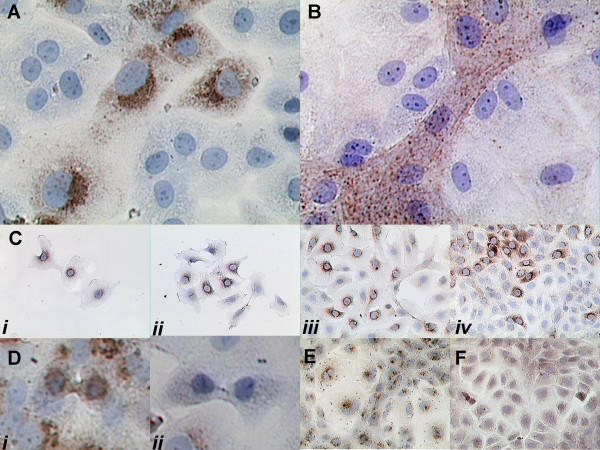
Immuno-characterization of IEC-18 cell heterotypy. **A**: Carboxyl IGFBP-2 immunostaining of the sequestered C2 fragment in cells with a preserved crypt cell phenotype but not in others. **B**: F-actin immunostaining (performed without antigen retrieval to visualize dynamic actin filaments) demonstrates intense staining in crypt cells but not in others. **C**: Cells plated at variable density with 10% FBS start out as weakly C2 positive (*i*) but have a progressive loss of C2 immunostaining prior to confluence (*ii-iv*). **D**: Immunolocalization of the C2 antigen demonstrates that both C2 positive (*i*) and C2 negative (*ii*) cells preserve their phenotypes during proliferation. **E-F**: Control immunolocalization using the IGF type 2 receptor (E) as a prelysosome-localized antigen and villin (F) as a cytoplasmic-localized antigen to demonstrate that both intravesicular and cytoplasmic antigens can be evenly detected throughout IEC culture.

We have recently discovered a novel antigen localization pattern by using anti-carboxyl IGF binding protein-2 (IGFBP-2) antibody (to an antigen which we call C2) to demonstrate a multivesicular pattern in some IEC-18 cells but not in others that are immediately adjacent (illustrated in Figure 1A). We note that anti-amino IGFBP-2 showed no staining in IEC-18 cells and that pretreatment with synthetic antigen abolished C2 staining (data not shown), confirming that C2 is a carboxyl fragment of IGFBP-2. For the purpose of this study, we wanted to determine whether or not this heterotypy represented cell fate divergence or systemic pleiomorphism.

To further characterize the observed heterotypy, we examined IEC-18 cells with anti-actin antibody in IEC-18 cells without performing antigen retrieval and allowing the detection reaction to proceed until only a limited amount of staining is seen. When used with the right antibody, this technique is a means to visualize dynamic portions of actin filaments, such as stress fibers and the proximal ends of microvilli, because they lack the actin binding proteins that obscure the antigen. If the reaction is allowed to continue, eventually all actin filaments will stain. In the version that we use, the assay is a qualitative assessment of cytoskeletal turnover when we are comparing two adjacent cells (because both cells had exactly the same conditions for detection of actin). Both stress fibers and microvilli are heavily stained and appear to have high turnover rates in cells that have C2 staining, but not in those without it (Figure 1B). We note that the punctate dots of filamentous-actin corresponded with small microvilli in C2 positive cells and that the C2 negative cells had a paucity of microvilli on their surface – which was confirmed by focusing up and down the shafts of the microvilli (data not shown).

### IEC-18 cells spontaneously lose C2 staining prior to confluence, despite 10% FBS

When IEC-18 cells were plated at variable densities in 10% fetal bovine serum (FBS), we saw that all crypt cells were initially positive for C2 staining, but with increasing density there were increasing numbers of C2 negative cells (Figure 1C). We also saw that C2 positive cells had increased staining intensity as they approached confluence. This experiment demonstrates two important points: first, that loss of C2 staining begins before confluence and second, that it can occur with comparable prevalence in the presence of FBS as it does in serum free media (SFM).

### C2 positive cells and C2 negative cells are both capable of phenotype preservation during proliferation

To determine if the C2 positive and negative IEC-18 cells were each capable of phenotype preservation during proliferation, IGFBP-2 stained cells on coverslips were searched for mitotic cells in the stage of cytokinesis and their images were captured by digital photomicroscopy (n = 6 cell pairs for each phenotype). In each case, all daughter cells had the same phenotype as their sister, confirming that both the C2 positive and C2 negative phenotypes are conserved in subsequent cycles of mitosis (representative examples in Figure 1D).

### C2 positive cell abundance is increased in proportion to the efficacy of IGF agonist treatment

**Figure 2:**
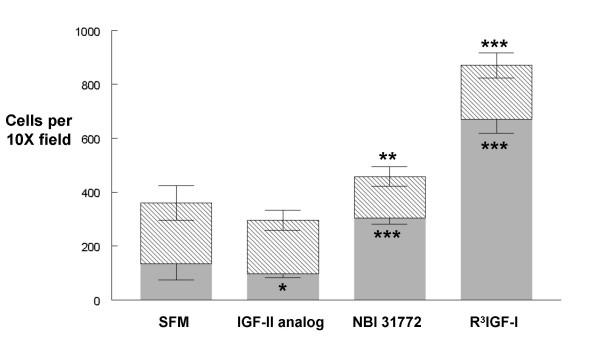
Mean number of C2 positive and C2 negative cells for each treatment condition. C2 positive cells are boxed in gray and C2 negative cells in stripes. Note that IGF-II analog reduces C2 positive cells when compared to SFM, whereas both NBI 31772 and R^3^-IGF-I significantly increase C2 positive and total cell abundance when compared to SFM (*** = p < 0.01, ** = p < 0.05, * = p < 0.1). In contrast, no treatment significantly altered C2 negative cell abundance.

Because IEC-18 cells grow best in the presence of high dose insulin, we suspected that crypt cell proliferation was dependent upon IGF receptor stimulation. In general, differentiated and benignly transformed epithelial cells are less likely to proliferate upon reaching confluence, so we sought to preferentially drive crypt cell proliferation in a graded fashion using different IGF receptor agonists (Figure 2). IGF-II analog, a weak agonist, reduced the crypt cell abundance while NBI 31772 (an agent that displaces IGFs from IGFBPs) increased it significantly and R^3^-IGF-I doubled the number of crypt cells per 10X field (also highly significant) while none of the treatments significantly altered the abundance of C2 negative cells. This strategy allowed us to systematically skew the cell composition and use gene array analysis to determine whether C2 positive cells were epigenetically divergent.

### Our gene array methodology identified four candidate genes as potential markers of cell fate divergence in IEC-18 culture

**Figure 3:**
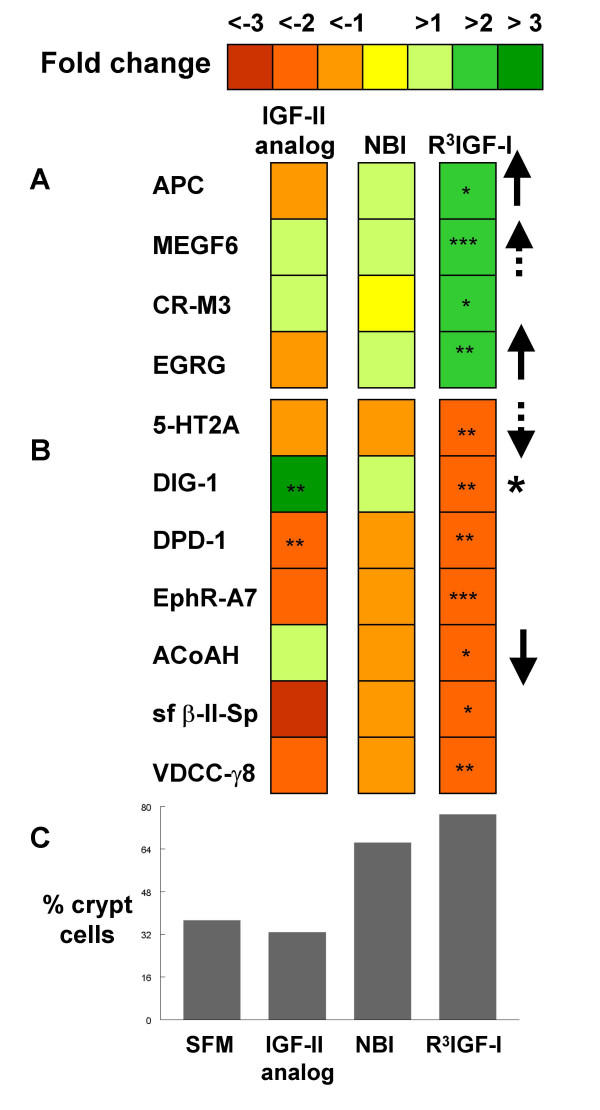
Gene array screen using skewed IEC-18 cell composition to find potential phenotypic markers. Affymetrix rat gene chips were used to compare RNA from SFM and R^3^-IGF-I treated cells to find individual genes with significant fold changes – defined as greater than two fold more (A) or two fold less (B) with a p-value of less than 0.1 for the purpose of this screen. These were then compared with the fold changes from IGF-II analog and NBI 31772 to look for fold change trends that paralleled the changing cell composition (summarized in C as the percentage of crypt cells). The results are presented as upward arrows (strong positive correlations), upward dashed arrows (weak positive correlations), downward arrows (strong inverse correlations) and downward dashed arrows (weak inverse correlations). The large asterisk points to an example where both direct receptor agonists resulted in a significant difference but NBI 31772 did not, suggesting a direct drug effect rather than an effect of cell composition – making this gene a less likely candidate. For smaller asterisks, *** = p < 0.01, ** = p < 0.05, and * = p < 0.1.

Eleven genes met our criteria for a significant fold change, four were positively correlated with crypt cell abundance and seven were inversely correlated (Figure 3). Of these first pass candidates, only six showed the appropriate fold trends across all treatment conditions, consistent with our hypothesis of constitutive expression that could reflect divergent cell fates. Of these six, one was found to have a significant difference between IGF-II analog and SFM, suggesting a direct treatment effect by IGF agonists but not by NBI 31772 (dithiolethione-inducible gene 1). Enzymatic glycosylation-regulating gene is known to be an insulin responsive gene and was also excluded because of the high probability of a direct effect by our IGF agonists [6]. Of the four remaining candidates, one (brain acyl CoA hydrolase) had absolute values that bordered on background levels in the SFM, IGF-II analog and NBI 31772 treatment conditions (defined as 10 arbitrary fluorescent units) and has had a relatively limited characterization in the literature [7-9]. We saw no obvious means for obtaining or generating an antibody to it and thought it unlikely to be a robust marker at the protein level, in large part because its message has only been found in brain thus far. Another had high homology with the EGF family and is currently a predicted protein based on genomic sequence [10]. However, the two remaining candidate genes we pulled out were well-characterized gut-related proteins (APC and 5-HT2A).

### Immunolocalization for 5-HT2A and APC revealed 2 divergent phenotypes within IEC-18 cell culture

**Figure 4:**
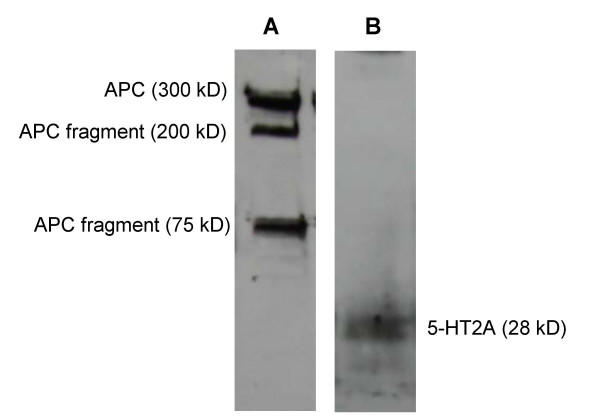
Western blots of APC and 5-HT2A in IEC-18 cell lysates. Visualization of the appropriate sized band (and break down products) for APC is shown in lane A and 5-HT2A is shown in lane B.

Western blots using antibodies against APC and 5-HT2A in IEC-18 cell lysates revealed staining of the appropriate sized band for each (Figure 4). In the case of APC, there were several discrete smaller bands which were inversely proportional in abundance to the 300 kD full-sized protein (consistent with proteolytic fragments) when compared across multiple samples (data not shown). In the case of 5-HT2A, there was a single 28 kD band that was faint, suggesting relatively low abundance. Both antibodies were deemed suitable for immunolocalization.

**Figure 5:**
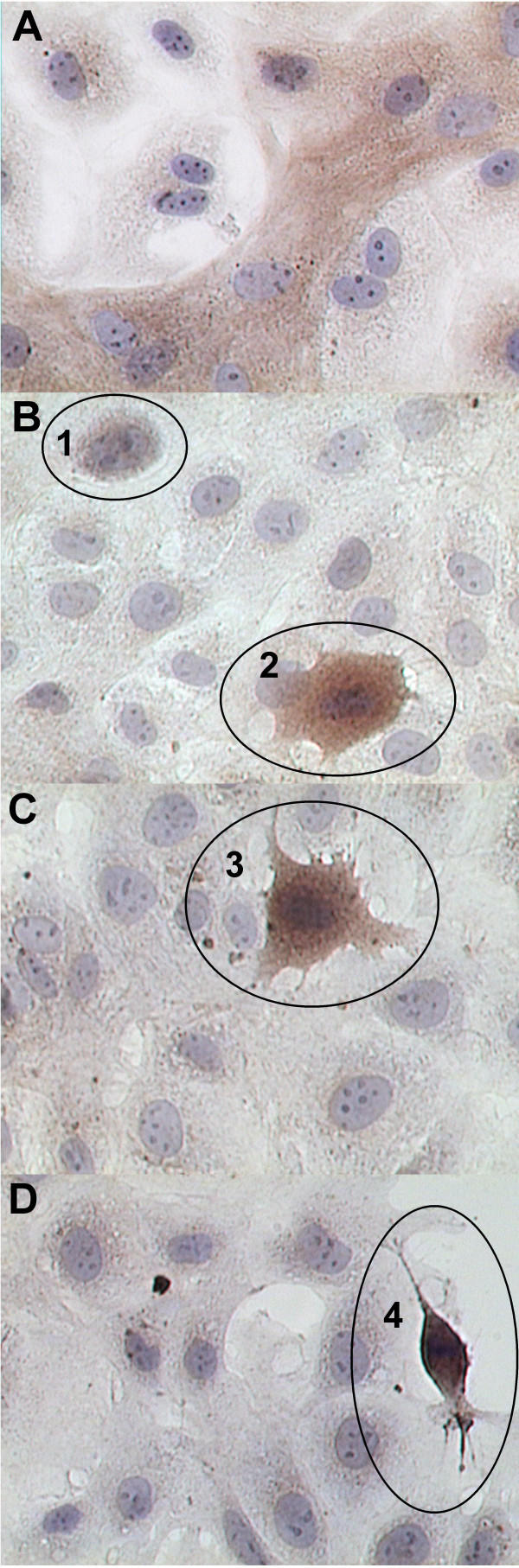
Immunolocalization of APC and 5-HT2A in IEC-18 culture. **A**: APC immunostaining parallels that of C2 and is found in crypt cell strands but is absent or scant in adjacent cells. **B-D**: 5-HT2A immunostaining is absent in the majority of cells but is found in a few rare cells. On closer inspection, we found that there seemed to be a progressive increase in staining intensity that correlated with a morphologic transition away from IEC-18 cell morphology and towards that of a neuroendocrine-like cell (illustrated by the numbered circles).

Immunolocalization for APC revealed that C2 positive cells also had intense APC staining whereas C2 negative cells either had limited or no APC staining (Figure 5A). This finding is in keeping with our gene array analysis and suggests that there is substantial and wide spread divergence of IEC-18 crypt cells away from the crypt cell phenotype and towards an adenoma-like transformation.

Immunolocalization for 5-HT2A demonstrated a second cell phenotype (Figure 5B,C and 5D). We had previously noted rare neuroendocrine-like morphology characterized by spindle-shaped cells with bipolar, dendritic arbors (unpublished findings), however with 5-HT2A immunostaining we found a mean of 5 positively stained cells per coverslip of confluent cells (n = 6 coverslips of confluent cells in SFM). 5-HT2A is present in high abundance within paneth and neuroendocrine cell types but is absent within small intestine crypt cells *in vivo *[11]. The cells that we observed appeared to be in transition, going from IECs to neuroendocrine-like cell morphology – with corresponding increases in staining intensity. While still a very low prevalence, the staining was quite intense in this small subset and was not detected in any of the adjacent cells.

### Double labeling confirms that C2 negative cells have diminished APC abundance

**Figure 6:**
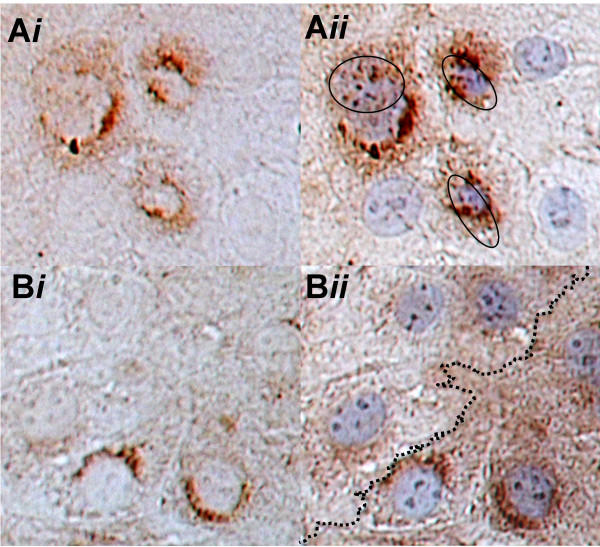
Double labeling overlay immunolocalization of C2 with either actin or APC. **A*i***. C2 immunostaining of wet-prepped cells. **A*ii***. C2 immunostaining in the same cells, overlaid with f-actin immunostaining. The proximal cores of actin-stained microvilli bundles located on C2 positive cells are encircled, whereas there are either no or few microvilli on C2 negative cells. **B*i***. C2 immunostaining of wet-prepped cells. **Bii**. C2 immunostaining in the same cells, overlaid with APC immunostaining. The dotted line divides C2 positive cells (below) from C2 negative cells (above). There is consistent APC staining in C2 positive cells, whereas there is variable and comparably reduced APC staining in C2 negative cells.

Double immunolabeling with overlay technique (using C2 and actin antibodies) demonstrated that C2 negative cells have a paucity of microvilli in comparison to C2 positive cells (Figure 6A). Additionally, double labeling with C2 and APC demonstrated that C2 positive cells have uniform APC staining whereas C2 negative cell have variable and overall diminished staining in comparison (Figure 6B). These experiments provide an objective demonstration that C2 positive cells retain the IEC phenotype whereas C2 negative cell are undergoing transformation (which is an obligatory step associated with the loss of APC).

## Discussion

In this manuscript we have taken advantage of an IEC immunolocalization marker that we call C2 to demonstrate two forms of cell fate divergence within IEC-18 culture. Using a combination of gene array screening and immunolocalization, we found that C2 is lost in over half the cells by the time of confluence and its loss is also associated with a down regulation of the APC gene, decreased APC protein abundance, decreased actin filament turn over, and reduced microvillar density. In short, there is an adenoma-like phenotype that fits with this genotype and this fits well with what is known about the function of the APC protein [12-15]. In addition, another cell genotype-phenotype correlate was detected by screening out the 5-HT2A gene and visualizing the cells that express its protein, i.e. neuroendocrine-like cells. These findings also fit well with cell phenotypes known to express 5-HT2A in the gut endoderm [11,16,17] and strongly argue against the dogma that IECs persist as a single lineage prior to reaching confluence.

While we think our findings have important implications for the existing IEC literature, the more important aspect of this manuscript may be in the methodology we have piloted. In many ways, cell culture, whether primary or immortalized, transformed or not, is by definition a model in flux. The progenitor lineage that a researcher starts with is rarely the hodge podge they end up with after a limited number of passages and it is common, if not expected that epigenetic drift will occur with each cell culture passage. However, what we describe in this manuscript is different in that IEC-18 cells are displaying a uniform trend in cell fate divergence – a trend that can be modulated with IGF. Many if not most gut epithelial cell lines are IGF (or high dose insulin) dependent for proliferation and thus are potentially vulnerable to this biological confounder. It will remain to be seen if other epithelial cell types have similar behaviors when examined in this fashion. Conversely, there is also a positive light to our findings; IEC-18 cells could be a compelling model for spontaneous adenomatous transformation because these adenoma-like cells are arising from a genetically competent progenitor prior to reaching confluence. To our knowledge, no such model with this property has been previously defined.

Our study has notable weaknesses and strengths. First, we have identified two divergent cell fates but have only partially characterized them because we were focused on developing a viable screening methodology (hence the ubiquitous use of the word "like" in this manuscript). Second, we are using a rat gene array chip that has approximately 9000 non-EST genes per chip. This is not an exhaustive survey of the rat genome and it is possible that there are other cell fates present in IEC-18 culture that we did not detect. Third, our phenotype assays are based on immunolocalization, which is a semi-quantitative technique with regards to assessing protein abundance. However, in this case we are actually combining cell-to-cell differences in protein abundance with distinguishing morphologic characteristics (e.g. loss of microvilli, flattening, bipolar shape, dendritic arbors, etc.) to delineate the phenotypes between adjacent cells. In short, what we are quantifying, in the case of adenoma-like cells, is the percentage of cells with a given phenotype. For this purpose, blinded immunolocalization is simple, quantitative and exceedingly efficient. The combined methodologies we chose result in a highly accurate technique for assessing divergence and their specificity can be bolstered by comparative studies of co-divergent markers (as we demonstrated with C2 and APC). As for other positives, the methodology is relatively rapid and can detect low prevalence phenotypes (as demonstrated by the anti-5-HT2A antibody). Additionally, we have demonstrated that a transcriptional marker is not required to create an effective screen. What is required, and what should probably prompt a researcher to employ this methodology, is a probe, a phenotype, or a pleiotropism that results in a consistently heterogeneous and quantifiable pattern (as C2 proved to be for us).

In closing, we point out one last caveat. Gene array investigation is an evolving science but there remain three potential pitfalls for every new application: experimental design flaws, data integrity issues and biological misassumptions [18-20]. In this study, we used a well-accepted screening principle (i.e. significant fold changes within individual genes in response to a treatment); we included a paired reference standard for each treatment condition (the SFM control); we increased the screening stringency by adding a requirement for parity in fold trend in accordance with changing cell compositions; and then confirmed our findings by phenotype assays. However, we demonstrated that a small minority of neuroendocrine-like cells were still able to significantly alter the outcome of our gene array screen (a possibility that we had thought to be remote, given our assay's stringency). We conclude that even low frequency epigenetic events can be a serious biologic confounder of gene array studies in cell culture.

## Conclusions

We have demonstrated a novel methodology for detecting and characterizing cell fate divergence in cell cultures derived from a common progenitor. The majority of IEC-18 cells are transformed into adenoma-like cells in SFM. IGF agonists reduce the rate of transformation by driving proliferation of the progenitor phenotype but do not prevent it. We also detected a very low incidence of differentiation toward a neuroendocrine-like cell type in these same cultures.

## Methods

### Cell culture

Rat ileal epithelial cells (IEC-18 – American Type Culture Collection, Rockville, MD) from aliquots of passage numbers 6–8 were grown to confluence in DMEM with 10% fetal bovine serum (FBS) and .1% insulin. The time period for complete epithelial cell confluence was 24–48 hours. Confluent cells were incubated for 72 hours in DMEM to establish a serum free period either alone or with 10^-6^M NBI 31772 (Calbiochem, San Diego, CA [21]), 0.5 × 10^-6^M IGF-II analog, or 0.5 × 10^-6^M R^3^-IGF-I (Sigma, St. Louis MO), as a means of boosting crypt cell proliferation. We point out that crypt cells can be driven to proliferate despite confluence whereas more differentiated epithelial cell types are resistant to IGF-driven proliferation upon reaching confluence. Alternatively, IEC-18 cells were diluted to 1/8, 1/4 and 1/2 the original seeding density and plated in DMEM with 10% FBS for 24 hours as a means to evaluate cell fate divergence in the presence of serum.

### Western blots

Cell lysate samples were collected from 150 mm dishes, washed with PBS and recovered in 700 uL of lysis buffer by scraping the dish. Cells were spun down for 10 minutes in a pre-chilled centrifuge. The supernatant was diluted 4:1 with loading buffer and run on 12 or 15% acrylamide gels. After the gel had been transferred onto a nitrocellulose membrane, 0.5% Ponceau S stain was applied to confirm equitable protein transfer across all lanes.

Western blot membranes were incubated in 5% milk block for 1 hour at RT. Goat polyclonal primary antibody for APC and 5-HT2A were applied for 1 hour at RT. Primary antibody was washed off with TBS-Tween and secondary antibody was placed on the membranes for one hour, then washed again. ABC (Elite Series, Vector Labs) was placed on the membrane for an hour and then washed and visualized with SuperSignal chemiluminescent substrate (Pierce, Rockford, IL), each was prepared per the manufacturer's instructions.

### Gene array analysis

IEC-18 cells were treated and processed as described in C*ell Culture *methods and then utilized to screen for specific cell fate markers. Total RNA was harvested for each treatment condition in triplicate experiments and then used for gene chip analysis per the manufacturer's protocol (rat gene chip # 230A, Affymetrix, Santa Clara, CA). All available non-EST tags were searched within the R^3^-IGF-I data set and those with significantly different fold changes when compared to SFM cells were selected as potential candidates for further analysis (for the purpose of a first pass screen, a significant fold change was defined as a p value < 0.1 and a greater than two fold increase or decrease). To further refine the list of potential candidates, the fold changes for NBI 31772 and IGF-II analog, which had step-wise reductions in effects upon crypt cell proliferation, were used to determine if there was a positive or inverse trend between a selected gene's mRNA abundance and crypt cell abundance. In this way, we sought to select constitutively expressed genes, whose primary differences in abundance would be due to differences in cell composition. Our statistical test for determining significance was a two-tailed paired t-test (provided as part of the standard analysis by the University of Virginia Biomolecular Research Facility and the Dept. of Health Evaluation Sciences [22]).

### Immunolocalization experiments

Immunolocalization in IEC-18 cells was performed for IGFBP-2 (C2), f-actin, IGF receptor type 2 and villin as well as two proteins that were screened out by our gene array analysis (APC and 5-HT2A) in a minimum of three separate experiments for each. In brief, the cells were fixed in formalin overnight, washed in DIG buffer (4% 1M Tris Base, 6% 5M NaCl, 16% 1M Tris HCl) 5 times then put in blocking solution (1% wt/vol BSA in DIG buffer) for 1 hour at room temperature. This was followed by a one-hour incubation with goat polyclonal primary antibody (all were obtained from Santa Cruz Biotechnology, CA; specifically for the anti-actin antibody the product number was sc-1615) in antibody diluent solution (5% 1M Tris HCl, 1% 1 M Tris Base, 9% NaCl, 3.3% Triton-X 100). Primary antibody was washed off with DIG × 5 and secondary antibody (donkey anti-goat [Jackson Immunoresearch Labs, West Grove, PA]) was placed on the slides for one hour. ABC (Elite Series, Vector Labs) was prepared and applied per the manufacturer's instructions, washed as above and visualized with DAB solution (Sigma, St. Louis, MO) in parallel. The slides were counterstained with hematoxylin, cover-slipped, photomicrographed and representative images were chosen for publication. With respect to the actin visualization, detection was performed such that colored precipitate was observed under the microscope and stopped when stress fibers and microvillus cores were evident and before the high background of the total cell f-actin began to stain efficiently. In the crypt cell quantification experiments, six 10X images were taken at random, printed on color paper at maximum size, blinded, and all cells were assessed as C2 positive or C2 negative and their means and standard deviations calculated for each treatment condition.

### C2 double labeling immunolocalization experiments

To objectively test our observations that C2 positive cells have a distinctive colocalization pattern when compared to C2 negative cells, C2 double labeling experiments with f-actin and with APC were performed as overlay experiments using digital microscopy. In brief, C2 immunolocalization was performed as described above except that a grid was drawn on the wet mount slide, allowing digital photomicrographs to be taken at 40X of C2 staining while still covered with stop solution and then a second antibody, either for f-actin or APC was applied and the same process repeated before being counter-stained with hematoxylin and cover-slipped. The same exact images were then re-mapped using the grid and the stored digital images were used for precise position verification. The overlaid double-labeled image was then taken and contrasted to the original image to allow comparison of differential localization of the two antigens.

## Abbreviations

*IGF*, insulin-like growth factor; *IGFBP-2*, IGF binding protein-2; *IGF-II analog*, synthetic truncated IGF-II; *R*^3^-*IGF-I*, synthetic long arginine IGF-I; *NBI 31772*, an alpha-numeric designation for a non-biologic compound that best displaced IGFs from IGF binding proteins in a large bioassay screen; *SFM*, serum-free media; *APC*, adenomatous polyposis coli; *5-HT2A*, serotonin receptor 2A; *C2*, IGFBP-2 carboxyl fragment

## Authors' contributions

PG designed the study, participated in the immunolocalization studies, and performed data analyses. JP carried out the immunolocalization, cell count, and Western blot studies. NF maintained the IEC cells lines and expertly provided uniformly confluent cells. PG and JP produced the figures and drafted the manuscript. All authors read and approved the final manuscript.
